# Accessory Gvp Proteins Form a Complex During Gas Vesicle Formation of Haloarchaea

**DOI:** 10.3389/fmicb.2020.610179

**Published:** 2020-11-12

**Authors:** Kerstin Völkner, Alisa Jost, Felicitas Pfeifer

**Affiliations:** Microbiology and Archaea, Department of Biology, Technical University of Darmstadt, Darmstadt, Germany

**Keywords:** protein-protein interaction, split-GFP, protein network, cellulose binding domain, *Haloferax volcanii*, *Halobacterium salinarum*

## Abstract

*Halobacterium salinarum* forms gas vesicles consisting of a protein wall surrounding a gas-filled space. The hydrophobic 8-kDa protein GvpA is the major constituent of the ribbed wall, stabilized by GvpC at the exterior surface. In addition, eight accessory Gvp proteins are involved, encoded by *gvpFGHIJKLM* that are co-transcribed in early stages of growth. Most of these proteins are essential, but their functions are not yet clear. Here we investigate whether GvpF through GvpM interact. Pull-down experiments performed in *Haloferax volcanii* with the cellulose-binding-domain as tag suggested many interactions, and most of these were supported by the split-GFP analyses. The latter study indicated that GvpL attracted all other accessory Gvp, and the related GvpF bound besides GvpL also GvpG, GvpH and GvpI. A strong interaction was found between GvpH and GvpI. GvpG showed affinity to GvpF and GvpL, whereas GvpJ, GvpK and GvpM bound GvpL only. Using GvpA for similar analyses yielded GvpF as the only interaction partner. The contact site of GvpF was confined to the N-terminal half of GvpA and subsequently mapped to certain amino acids. Taken together, our results support the idea that the accessory Gvp form a complex early in gas-vesicle assembly attracting GvpA *via* GvpF.

## Introduction

Halophilic archaea (haloarchaea) thrive in hypersaline environments such as salt lakes or salterns containing up to 30% NaCl. They adapt to these salty conditions by maintaining a similarly high KCl concentration in the cytoplasm. The haloarchaea *Halobacterium salinarum* and *Haloferax mediterranei* produce gas vesicles allowing the cells to float to the surface of the brine. These gas-filled vesicles consist of a wall exclusively formed by proteins. Major component is the hydrophobic, 8-kDa GvpA aggregating into a low-pitch helix seen by transmission electron microscopy as 4.6 nm wide ribs running perpendicular to the long axis of the gas vesicle (Walsby, 1994; Offner et al., 1998; Pfeifer, 2012). The protein wall is stabilized on the exterior surface by the second structural protein, GvpC (Englert and Pfeifer, 1993). Gases are able to diffuse in and out, presumably *via* small holes in the wall. Water vapor might enter the hollow space, but is unable to precipitate because of the hydrophobic interior surface of the wall (Walsby, 1994). Electron microscopic studies of haloarchaeal cells indicate small bicones already filled with gas in early stages of growth; these bicones are enlarged to spindle- or cylinder-shaped structures. The final diameter of the haloarchaeal gas vesicle is 200–250 nm; and the cylinder-shaped structures can grow as long as 2 μm (Walsby, 1994; Knitsch et al., 2017).

Gas vesicles of haloarchaea are encoded by a gene cluster consisting of 14 gas vesicle protein (*gvp)* genes arranged as *gvpACNO* and *gvpDEFGHIJKLM*, and eight of these genes are essential to produce a gas-filled particle as determined in *Haloferax volcanii* transformants (Englert et al., 1992; Offner et al., 2000). This moderately halophilic haloarchaeon is easy to transform, grows faster than *Hbt. salinarum* and lacks all of the *gvp* genes. The six non-essential Gvp proteins are the surface-attached GvpC, the two regulatory proteins GvpD and GvpE (Krüger et al., 1998; Zimmermann and Pfeifer, 2003; Hofacker et al., 2004), and the proteins GvpH, GvpI and GvpN (Offner et al., 2000). The absence of GvpC in *Hfx. volcanii* ΔC transformants (the ΔC construct contains except *gvpC* all *gvp* genes) leads to long and weak gas vesicles with altering diameters (Offner et al., 1996), whereas the absence of GvpD and GvpE causes a low expression of the *gvp* gene cluster. In the case of GvpH and GvpI altered gas vesicle structures are observed. ΔH transformants produce weaker gas vesicles of wild type shape, whereas extremely long and cylinder-shaped gas vesicles are found in ΔI transformants. The lack of GvpN leads to small bicones that are not enlarged (Offner et al., 2000). A deletion of any other *gvp* gene in the cluster leads to gas vesicle negative (Vac^–^) transformants (Offner et al., 2000). Isolated gas vesicles cannot be disintegrated into their protein constituents and separated by sodium dodecyl sulfate polyacrylamide gel electrophoresis (SDS-PAGE); only GvpC is washed off and separated on the gel (Englert and Pfeifer, 1993). Aggregates of the hydrophobic GvpA dissolve in 80% formic acid only, and dialysis to remove the formic acids leads to amorphous precipitates of GvpA (Belenky et al., 2004). The protein constituents of the gas vesicles were determined by MS/MS-based proteome analyses or immunological methods (Shukla and DasSarma, 2004; Chu et al., 2011). Except for GvpD, GvpE, and GvpK, all Gvp proteins are present in gas vesicle preparations suggesting that they are constituents of the wall or are attached to the structure during gas-vesicle assembly.

The transcript encoding the accessory gas vesicle proteins GvpF through GvpM occurs in early exponential growth, implying that these proteins are required in early stages of gas-vesicle assembly (Offner and Pfeifer, 1995). Putative interactions of GvpM have been studied by affinity chromatography with His-tagged Gvp and Ni-NTA matrices, and the results suggest that GvpM is able to bind GvpH, GvpJ and GvpL, but not GvpG (Tavlaridou et al., 2014). For these analyses, _His_Gvp proteins were synthesized in *Escherichia coli*, purified under denaturing conditions and refolded in high salt solutions. Haloarchaea require 15–30% NaCl for growth, and adapt by maintaining a similarly high KCl concentration in the cytoplasm. The haloarchaeal proteins are usually adapted to these salt concentrations and often denature in low salt solutions. Thus, the purification of these proteins from *E. coli* in low-salt solutions might have influenced the protein structure and interaction. Also, other histidine-rich haloarchaeal proteins bind non-specifically to the Ni-NTA or Ni-sepharose matrices used for the binding of His-tagged Gvp to select putative binding partners and complicate the analysis.

To study putative interactions of Gvp proteins in high salt, we recently adapted the split-GFP method (Ghosh et al., 2000; Magliery et al., 2005) to high salt solutions and studied protein-protein interactions in *Hfx. volcanii* transformants (Winter et al., 2018). We used a derivative of the salt-adapted green fluorescent protein smRS-GFP (Reuter and Maupin-Furlow, 2004) with higher fluorescence, mGFP2 (Born and Pfeifer, 2019). The mGFP2 protein was split in the N- and C-terminal fragments (NGFP and CGFP) that were fused to the two proteins of interest. Both fragments do not assemble *in trans*, but will form a fluorescent GFP when the two fusion partners interact. The fluorescence of the transformants can be easily quantified (Winter et al., 2018). For each protein, four different N/CGFP fusions (N- or CGFP fused N- or C-terminally) are produced and eight combinations tested per protein pair to exclude putative steric hindrances for the assembly of GFP. Using this method, the interaction of GvpL/GvpM was confirmed, and the interaction site in GvpM confined to the N-terminal 25-amino acid (aa) (Winter et al., 2018). GvpM also interacted with GvpF and GvpH, and both proteins bound to the C-terminal 25 aa of GvpM (Winter et al., 2018).

In this report, we present a comprehensive study on the interactions of the accessory proteins GvpF through GvpM. Two different methods were applied, i.e., pull-down experiments with the cellulose-binding domain, CBD, and the *in vivo* analysis by split-GFP. The cellulose binding domain derives from *Clostridium thermocellum* and is part of the CipB protein (Poole et al., 1992; Morag et al., 1995). Proteins tagged with CBD can be selected by cellulose in high salt solutions (Ortenberg and Mevarech, 2000; Irihimovitch and Eichler, 2003; Schlesner et al., 2012). The _CBD_Gvp proteins and their putative Gvp binding partners were selected from lysates of *Hfx. volcanii* producing both bait and prey proteins. Each of the accessory Gvp proteins interacted with other Gvp, and _CBD_GvpM attracted all of them at once. In addition, each protein pair was tested by split-GFP in *Hfx. volcanii* transformants, and an interaction network was deduced. GvpL and GvpF had several interaction partners, whereas GvpH and GvpI bound each other and also interacted with GvpF and GvpL. In addition, the major gas vesicle structural protein GvpA was tested by split-GFP for interactions with these accessory proteins, and GvpF appeared to be the only binding partner. More detailed analyses with fragments of GvpA and variants of GvpA carrying single substitutions confined the interaction site of GvpF in GvpA.

## Materials and Methods

### Strains and Cultivation Conditions

The *Escherichia coli* strains One Shot Top10 (Invitrogen by Life Technologies) and GM1674 (*dam*^–^) (Palmer and Marinus, 1994) were grown in Luria-Bertani media at 37°C overnight. For the selection of transformants, 100 μg/mL ampicillin was added. The haloarchaeon *Haloferax volcanii* WR340 was grown in salt media containing 3 M NaCl, 150 mM MgSO_4_, 50 mM KCl, 0.05% (w/v) CaCl_2_, 25 mM Tris–HCl pH 7.2, 10 nM MnCl_2_, 0.5% (w/v) tryptone, 0.3% (w/v) yeast extract and 0.02% (w/v) histidine. For solid media, 1.8% (w/v) agar was added. To select *Hfx. volcanii* transformants, 6 μg/mL mevinolin (for selection of pWL_fdx_) and 0.2 μg/mL novobiocin (for selection of pJAS35) were added. Cultures on solid medium were incubated at 42°C for 3–7 days in a humid atmosphere. Liquid cultures were grown for 3–5 days on a shaker at 180 rpm and 42°C. Cultures forming GFP were initially incubated at 37°C for 24 h before they were transferred to 30°C overnight to enhance fluorescence, always shaking at 180 rpm.

### Vector Construction and Transformation of *Hfx. volcanii*

The split-GFP shuttle vectors pJAS-NGFP-Nterm and -Cterm, as well as pWL-CGFP-Nterm and -Cterm have been described previously (Winter et al., 2018). They are based on the two compatible vector plasmids pJAS35 (Pfeifer et al., 1994) and pWL_fdx_ (Scheuch and Pfeifer, 2007). The salt adapted mGFP2 is split between the residues 157 and 158, resulting in the NGFP and CGFP fragments. Nterm describes a fusion of the respective *mgfp2* fragment to the 5′-terminus of the gene of interest, whereas Cterm stands for a fusion at the 3′-terminus. The respective *mgfp2* fragments plus linker region are present in these vectors. The size of the linker encoded by the pJAS-derived shuttle vector is 14 aa [(GGSGSGS)_2_], whereas the linker derived from the pWL_fdx_ vector is 16 aa long [(GGSG)_4_] (Winter et al., 2018). The *gvp* reading frame under investigation was fused to *ngfp* or *cgfp* by inserting the respective fragment in these vector plasmids. The *gvp* reading frames were amplified by PCR using the p-vac region as template (containing the 14 *gvp* genes of *Hbt. salinarum* PHH1; Englert et al., 1992), and oligonucleotides including the desired restriction site for insertion (Supplementary Table 1). The *Nco*I-*gvp-Blp*I fragments amplified were inserted in pJAS-NGFP-Nterm or pJAS-NGFP-Cterm. For the insertion of *gvp* in pWL-CGFP-Nterm, *BamH*I and *Kpn*I sites were used, and *Nco*I and *BamH*I for the insertion in pWL-CGFP-Cterm. In some cases, *Bsp*HI (*gvpF*, *gvpI gvpJ*, *gvpL*) or *Pci*I (*gvpG*), was used instead of *Nco*I. Due to the presence of a *Kpn*I site in *gvpH*, the amplified *gvpH* fragment was blunt-ended inserted in pWL-CGFP-Nterm.

For pull-down assays of CBD-tagged proteins, the shuttle vector pCBD was used (Supplementary Figure 1). The *cbd* reading frame encodes the cellulose binding domain of the CipB protein from *Clostridium thermocellum* (Poole et al., 1992; Morag et al., 1995). The *cbd* reading frame was amplified from plasmid pWL-CBD-sec11b (Fine et al., 2006) and inserted in pWL_fdx_ *via Nco*I and *Kpn*I. Two additional cloning sites are present (*Bam*HI, *Xba*I) allowing the insertion of the respective *gvp* reading frame upstream or downstream of *cbd* [*Nco*I and *Bam*HI upstream of *cbd* are used to yield X_CBD_, and *Xba*I and *Kpn*I downstream to yield _CBD_X (with X = GvpF, G, H, I, J, K, L, or M)]. The expression of the respective gene fusion is under control of the strong ferredoxin promoter, *P*_*fdx*_ (Pfeifer et al., 1994). The *gvp* sequences were amplified using p-vac as template. The oligonucleotides used are listed in Supplementary Table 1. For the construction of pF-L^ex^, the *gvpFGHIJKL* reading frames of p-vac were amplified as *Xba*I–*Kpn*I fragment and inserted in pJAS35 for expression under *P*_*fdx*_ control.

In all cases, the correct insertion was verified by DNA sequence analysis. To avoid a restriction barrier in *Hfx. volcanii* WR340, the plasmid DNA was demethylated by passage through *E. coli* GM1674 (*dam*^–^) (Palmer and Marinus, 1994). *Hfx. volcanii* was transformed simultaneously with the two vector plasmids as described previously (Pfeifer and Ghahraman, 1993). In each case, the presence of both plasmids was confirmed by PCR, and the presence of the respective Gvp protein determined by Western analysis.

### Western Analysis

To verify the expression of *gvp-gfp* or *gvp-cbd* fusions, total protein was isolated from 50 mL cultures of the transformants in the late exponential growth phase. Cells were harvested by centrifugation (2,370 × *g*, 30 min, 4°C) and re-suspended in 5 mL lysis buffer (2.5 M KCl, 50 mM MgCl_2_, 1 mM EDTA, 5% (v/v) glycerol, 50 mM Tris–HCl pH 8.0) + DNaseI (0.1 mg/mL). Cells were lysed by sonification on ice. The lysate was cleared from cell debris by centrifugation and dialyzed against 10 mM Tris–HCl pH 7.2 overnight at 20°C to eliminate salts. 20 μg of total protein was separated by SDS-PAGE (Schägger and von Jagow, 1987) and transferred to a PVDF membrane (Roti Fluoro PVDF, Carl Roth) using a Semidry-Blotter (PerfectBlue^TM^, 30 min, 2 mA). The membrane was dried for one hour, reactivated with 100% (v/v) methanol and washed two times in PBS (137 mM NaCl, 2.7 mM KCl, 10 mM Na_2_HPO_4_, 2 mM KH_2_PO_4_) before blocking for an hour with Odyssey Blocking Buffer (Licor). The membrane was incubated with the respective antiserum raised against GvpF, GvpG, GvpH, GvpI, GvpJ, GvpK, GvpL, GvpM, or isolated gas vesicles (to detect GvpA). The membrane was washed four times in PBS + 0.1% (v/v) Tween20, incubated with the secondary antibodies labeled with IRDye 800 CW (Licor) for 1–2 h at 20°C, and washed four times for 5 min in PBS + 0.1% (v/v) Tween20. Excessive Tween20 was removed by washing the membrane with PBS. The fluorophore coupled to the secondary antibody is detectable at 800 nm with the Odyssey Fc Imager (Licor).

### Quantitation of GFP Fluorescence

The fluorescence of *Hfx. volcanii* transformants was measured to determine the formation of fluorescent GFP as a result of an interaction of the two fused proteins of interest. Transformants were grown as described to OD_600nm_ 1.5–2. Two mL of the culture were harvested (2 min, 9,600 x *g*, 20°C), the cells washed in 1 mL basal salts (3 M NaCl, 150 mM MgSO_4_, 50 mM KCl), and re-suspended in 500 μL basal salts. To investigate equal amounts of cells, the OD value was adjusted to OD_600nm_ 1, and 300 μL of the culture transferred into a microtiter dish. For each Gvp protein combination, two biological and three technical replicates were investigated. The fluorescence was measured (Phosphorimager FLA-5000 and the software Fujifilm science lab image gauge ver. 4.24), and the fluorescence intensity obtained in light absorbing units (LAU) per mm^2^. The relative fluorescence, rf, was calculated as described (Winter et al., 2018). The *p*-value was determined by Student’s *T*-test.

### Affinity Chromatography Using CBD-Tagged Proteins

Each accessory Gvp was fused to the cellulose binding domain, CBD, at the N- or C-terminus (_CBD_X or X_CBD_) and lysates of the respective _CBD_X/Y transformants were tested in pulldown experiments (X, Y = any accessory Gvp). In the case of _CBD_M, also _CBD_M/pF-L^ex^ transformants were tested. For each pull-down experiment, 400 mL cultures were grown at 37°C, 180 rpm to the late exponential growth phase. The cells were harvested (12,210 × *g*, 20 min, 4°C), and resuspended in 5 mL lysis buffer (2.5 M KCl, 50 mM MgCl_2_, 1 mM EDTA, 5% (v/v) glycerol, 50 mM Tris–HCl pH 8.0) plus DNaseI (0.1 mg/mL). The cells were lysed by ultrasound (Branson Sonifier 250, duty cycle: 55%, output control: 5, 2 min) and the suspension centrifuged for 20 min at 2,370 × *g*, 4°C. The soluble protein fraction (7 mL) was incubated with 1 mL of a 10% (w/v) cellulose suspension (Avicel PH-101, Sigma Aldrich) for 30 min at room temperature on an overhead rotator. The suspension was centrifuged (2,370 × *g*, 30 s), the supernatant removed and the resulting cellulose pellet with bound proteins resuspended in 600 μL washing buffer (2.5 M KCl, 50 mM Tris–HCl pH 8.0). The solution was transferred to an empty Mobicol column (Mobitec) and centrifuged (1,000 × *g*, 1 min), followed by six washing steps with 600 μl washing buffer. For protein elution, the cellulose was resuspended in 500 μL 100% ethylene glycol, incubated for 1 min at room temperature and centrifuged at 4,700 × *g*, 5 min. All fractions were dialyzed against 10 mM Tris–HCl, pH 7.2 overnight. For the analysis of proteins, 15 μL of each fraction were separated by SDS-PAGE (Schägger and von Jagow, 1987). The Gvp proteins were detected by Western analysis using the respective antisera.

## Results

Two different methods were applied to investigate protein-protein interactions. The first approach was pull-down experiments using the cellulose-binding domain as tag, and the second approach was split-GFP to identify interaction partners *in vivo*.

### Pull-down Experiments Using the Cellulose Binding Domain

Putative interactions of the accessory proteins GvpF through GvpM were investigated by pull-down experiments using the cellulose binding domain, CBD, allowing tagged proteins to bind cellulose at high salt concentrations (2–3 M KCl) (Ortenberg and Mevarech, 2000; Irihimovitch and Eichler, 2003; Schlesner et al., 2012). CBD was fused to the N- or C-terminus of each accessory Gvp using the vector plasmid pCBD (Supplementary Figure 1). The resulting _CBD_X or X_CBD_ constructs (X = any accessory GvpF through GvpM) were used to transform *Haloferax volcanii* WR340. To test whether the CBD-fusion proteins can be indeed purified in 2 M KCl, lysates of the respective _CBD_X transformants were mixed with cellulose to bind the CBD-tagged Gvp, and the elution fractions were investigated by SDS-PAGE as well as Western analysis using antisera raised against the various Gvp proteins (Supplementary Figure 2). Except for GvpJ and GvpM, the accessory Gvp were isolated in decent amounts (Table 1), and well visible in the Coomassie-stained gels (Supplementary Figure 2). GvpJ and GvpM were obtained in much lower amounts, presumably due to their hydrophobic nature and tendency to aggregate. Aggregates of GvpJ and GvpM might be present in the solid fraction of the cell extracts, and the addition of the detergents DDM (n-dodecyl-β-D-maltoside) or OGP (octyl-β-D-glucopyranoside) to the lysate and wash buffer slightly improved the presence of these proteins in the soluble fraction (Table 1). All of the accessory Gvp proteins including GvpJ and GvpM were detectable by Western analysis (Supplementary Figure 2). A smear of larger bands is always detectable with GvpJ, indicative of its ability to aggregate (Supplementary Figure 2). Thus, each of the _CBD_Gvp proteins was selected and could be purified. To ensure that the untagged Gvp interacted neither with the cellulose matrix nor with CBD, transformants were produced harboring the plasmids pCBD (without *gvp*) and pX^ex^ (*gvpX*-pJAS35 expressing any *gvp* without *cbd* fusion). None of the Gvp proteins tested was detectable in the respective elution fraction demonstrating that none of them bound to cellulose or to CBD itself (data not shown).

**TABLE 1:** Amount of _CBD_Gvp recovered from a 400 ml culture by CBD.

**Protein**	Amount in μg*
_CBD_F	146 ± 44
_CBD_G	307 ± 41
_CBD_H	417 ± 86
_CBD_I	142 ± 18
_CBD_J	23 ± 5
_CBD_K	214 ± 35
_CBD_L	484 ± 21
_CBD_M	13 ± 2
_CBD_J (1.4 mM DDM)	67 ± 14
_CBD_J (10.4 mM DDM)	132 ± 20
_CBD_J (60 mm OGP)	90 ± 9
_CBD_M (1.4 mM DDM)	14 ± 0
_CBD_M (10.4 mM DDM)	33 ± 14
_CBD_M (60 mM OGP)	18 ± 5

* The standard deviation was calculated from two biological and three technical replicates.

The CBD-tagged Gvp proteins were used as bait and tested for the selection of a putative Gvp interaction partner produced in the same cell. The first protein pair tested was GvpL/GvpM, where an interaction has been demonstrated using His-tagged Gvp proteins. GvpL or GvpM carried CBD fused to the N- or C-terminus (_CBD_L, L_CBD_ or _CBD_M, M_CBD_), and the combinations _CBD_L/M, L_CBD_/M, _CBD_M/L and M_CBD_/L were analyzed. Lysates of the _CBD_L/M and L_CBD_/M transformants were tested for the presence of GvpM, and monomers and dimers of this protein were identified by Western analysis (Supplementary Figure 3). Also, GvpL was present in the elution fractions of the _CBD_M/L and M_CBD_/L transformants indicating that GvpL bound GvpM (Supplementary Figure 3). These results confirmed the GvpL/GvpM interaction already seen using the respective His-tagged Gvp proteins and a Ni-NTA matrix for affinity chromatography (Tavlaridou et al., 2014).

**FIGURE 1:**
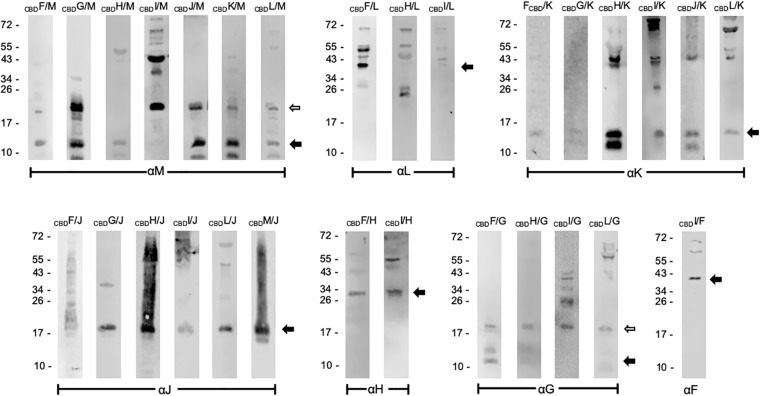
Western analyses of the pull-down assays using _CBD_X. The bait _CBD_X and the prey protein Y (X, Y = any GvpF through GvpM protein) were synthesized in the same *Hfx. volcanii* cell. Both are marked on the top of the blots. Only the elution fraction of the cellulose matrix is shown. In each case, 15 μL of the elution fraction was separated by SDS-PAGE, the proteins transferred to a PVDF membrane and incubated with the respective antiserum indicated underneath (αF through αM). The putative interaction partners were visualized with the fluorophore-labeled secondary antibody IRDye 800 CW (Licor). The blots are inverted to black-white and arranged according to the respective antiserum used. Arrows mark the expected Gvp monomer and dimer. Numbers on the left are size markers in kDa.

Similar pull-down experiments with CBD were performed with all other accessory Gvp proteins. The respective *Hfx. volcanii* transformants always carried two constructs, one for the bait protein (_CBD_X), and one for the prey GvpY (with X, Y = F, G, H, I, J, K, L, or M). Both reading frames encoding bait and prey were expressed under *P*_*fdx*_ promoter control to ensure a similarly high expression. Not all possible interactions were performed in both ways (_CBD_X+Y and _CBD_Y+X), since protein interactions were already found with one protein pair. The results of these studies are presented in Figure 1 and summarized Table 2. The Western blots in Figure 1 are arranged according to the antiserum used to detect the prey protein. Using _CBD_X to select GvpM yielded monomeric GvpM with _CBD_H, and monomers as well as dimers with _CBD_F, _CBD_G, _CBD_J, _CBD_K, and _CBD_L (Figure 1). Dimers and multimers of GvpM were observed with _CBD_I (Figure 1). Thus, GvpM was pulled-down in all these cases. GvpL was selected by _CBD_F, but the results obtained with _CBD_H or _CBD_I were ambiguous (Figure 1). However, when _CBD_L was used as bait, GvpM, GvpK, GvpJ, or GvpG were selected again suggesting that all these accessory Gvp bound GvpL. Also, GvpK interacted with any of the accessory Gvp, since _CBD_F through _CBD_J and _CBD_L pulled-down GvpK, and _CBD_K recovered GvpM. Monomers of GvpJ were selected by _CBD_X, but a smear including additional GvpJ multimers were found with _CBD_F, _CBD_H, _CBD_I, _CBD_L, or _CBD_M (Figure 1). GvpI was only used as bait, and _CBD_I pulled-down any of the accessory Gvp proteins. It is interesting to note that _CBD_I often induced the formation of larger oligomers, especially with GvpM, GvpK or GvpJ as prey. GvpH was selected by _CBD_F or _CBD_I, and _CBD_H pulled down GvpG, GvpJ, GvpK, GvpL or GvpM (Figure 1 and Table 2). GvpG monomers and dimers were selected by _CBD_F, whereas _CBD_H, _CBD_I and _CBD_L selected the dimer of GvpG (and additional multimers in the case of _CBD_I) (Figure 1). Since _CBD_G bound GvpM, GvpK and GvpJ, these results demonstrated that GvpG is able to interact with all accessory proteins. Similar results were obtained with GvpF, since GvpF was pulled down by _CBD_I, and _ CBD_F pulled down GvpG, GvpH, and GvpJ through GvpM (Figure 1 and Table 2). Overall, the results of these pull-down experiments implied that any accessory Gvp had multiple interaction partners and suggested that these proteins might form a larger complex.

**TABLE 2:** Summary of the pull-down experiments using _CBD_Gvp.

	GvpF	GvpG	GvpH	GvpI	GvpJ	GvpK	GvpL	GvpM
_CBD_F		GvpG	GvpH	+	(GvpJ) (aggregate)	GvpK (weak)	GvpL	GvpM
_CBD_G	+		+	+	(GvpJ) (monomer+dimer)	GvpK (weak)	+	GvpM
_CBD_H	+	GvpG (dimer)		+	(GvpJ) (aggregate)	GvpK	(GvpL)	GvpM (monomer)
_CBD_I	GvpF	GvpG (dimer + multimer)	GvpH	+	(GvpJ) (monomer+ aggregate)	GvpK (Monomer+ multimer)	(GvpL)	GvpM (dimer + multimer)
_CBD_J	+	+	+	+		GvpK	+	GvpM
_CBD_K	+	+	+	+	+		+	GvpM
_CBD_L	+	GvpG (dimer)	(−)	(−)	GvpJ (monomer)	GvpK		GvpM (monomer)
_CBD_M	+	+	+	+	GvpJ (aggregate)	+	+	

*GvpX interaction detected. + interaction was shown in another combination. − no interaction identified. ( ) interaction not clear.*

### Accessory Gvp Proteins Selected by _CBD_M

**FIGURE 2:**
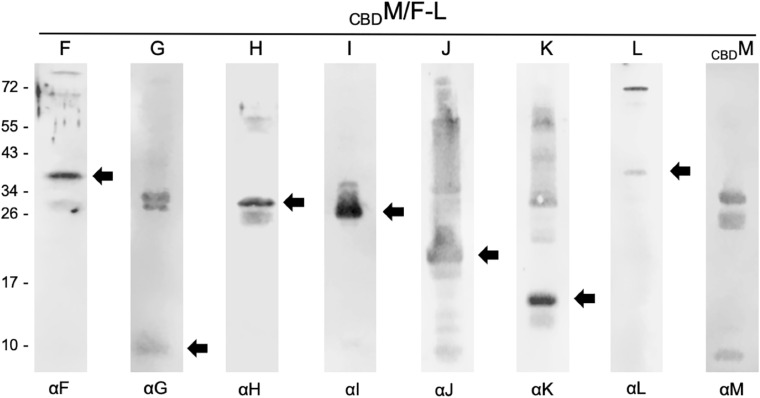
Western analyses of proteins selected by _CBD_M in _CBD_M/pF-L^ex^ transformants. In each case, 15 μL of the elution fraction was separated by SDS-PAGE, transferred to a PVDF membrane and incubated with the respective antiserum marked underneath (αF through αM). Reactions are visualized with the fluorophore-labeled secondary antibody IRDye 800 CW (Licor). Arrows mark the expected Gvp monomer detected. The blots were inverted to black-white. Numbers on the left side are size markers in kDa.

To investigate whether GvpM was able to select all accessory Gvp at once, *Hfx. volcanii* transformants were produced carrying _CBD_M and in addition the plasmid pF-L^ex^ expressing *gvpFGHIJKL* under the strong *P*_*fdx*_ promoter control. The interaction partners of _CBD_M were selected by pull-down experiments from the lysate of this transformant, and samples were tested by Western analyses using the different antisera (Figure 2). All accessory Gvp were identified, implying that _CBD_M attracted all of them. In each case the respective monomeric Gvp was detected. In the case of GvpG, two 30–34 kDa bands were visible in addition to the expected 10-kDa GvpG protein, and GvpJ and GvpK also formed multimers (Figure 2). The monomer of the _CBD_M bait protein was also detected (Figure 2). Overall, _CBD_M was able to pull down GvpF through GvpL. It is possible that each of these Gvp proteins bound independently to _CBD_M, but it is also possible that some or all of them formed a complex that bound to GvpM.

### Protein-Protein Interactions Investigated by Split-GFP

**FIGURE 3:**
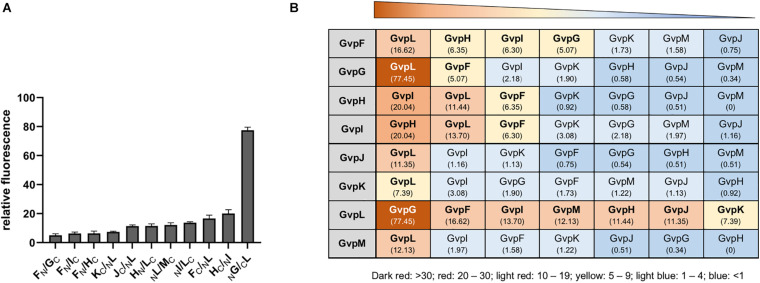
Interactions of the accessory Gvp determined by split-GFP. **(A)** Highest relative fluorescence (rf-values) determined for the protein-protein interactions calculated as described in the methods section. All experiments were performed with two biological and three technical replicates. **(B)** Summary of the different protein-protein interactions determined by split-GFP. The Gvp proteins tested are shown in the gray box on the left and their interaction partners are arranged according to the highest rf-value determined (given underneath). A fluorescence exceeding rf > 5 was regarded as clear interaction. Rf-values < rf 4 are regarded as weak or no interaction.

Pairwise interactions of the accessory Gvp proteins were studied in *Hfx. volcanii in vivo* using the split-GFP method. Each *gvp* reading frame was amplified by PCR and inserted in the four vector plasmids to fuse the *ngfp*- or *cgfp* reading frame to the 5′ or 3′ terminus of each *gvp* (Winter et al., 2018). The N- and C-terminal GFP fragments NGFP and CGFP derive from the salt-adapted, green fluorescent protein mGFP2 (Born and Pfeifer, 2019). A fluorescent GFP is only formed when the two fusion partners interact and steric hindrance does not occur. The reading frame of each fusion protein is expressed under the *P*_*fdx*_ promoter control to yield similarly large amounts of these proteins. The four N/CGFP fusions were designated _N_X, or _C_X for the N-terminal fusion, and X_C_ or X_N_ for the C-terminal fusion with Gvp (with X = respective accessory Gvp; C = CGFP; N = NGFP). The eight combinations of a protein pair were tested in *Hfx. volcanii* transformants and the fluorescence was measured in arbitrary light absorbing units per mm^2^. The highest relative fluorescence (rf-values) calculated for each interaction pair are shown in Supplementary Figure 4, and the original data obtained in LAU/mm^2^ are presented in Supplementary Figure 5. A summary of these data is shown in Figure 3.

The highest relative fluorescence of all interactions was observed with the _N_G/_C_L transformant (rf 77.5) implying a strong interaction of GvpL with GvpG (Figure 3A). All other interaction partners of GvpL yielded rf-values below 20. Rf-values between 10 and 20 were observed for the interaction F_C_/_N_L, _N_I/L_C_, M_C_/L_N_, H_N_/L_C_, and J_C_/_N_L, and rf 7.4 was determined for K_C_/_N_L (Figures 3A,B and Supplementary Figure 5). These results implied that the 32-kDa GvpL interacted with all other accessory Gvp. GvpL is the largest of the accessory Gvp and might act as platform to bind all others. Also, GvpF interacted with several Gvp proteins (GvpL, GvpH, GvpI, and GvpG) (Figure 3B). The F_C_/L_N_ transformant (rf 16.6) exhibited the highest relative fluorescence, whereas the other three binding partners yielded lower rf-values (rf 5.0–6.3). In the case of GvpF it should be mentioned that the highest fluorescence was always observed when N- or CGFP was fused to the C-terminus of GvpF, suggesting that a fusion to the N-terminus hinders the assembly of mGFP2. The 23-kDa GvpF is smaller than GvpL, but both proteins exhibit sequence similarities and a similar 3D-structure when modeled using the crystal structure of the cyanobacterial GvpF as template (Xu et al., 2014; Winter et al., 2018).

The second highest fluorescence (rf 20) of all combinations was observed for the H_C_/_N_I transformant (Figure 3), implying that GvpH and GvpI attract each other. Both proteins also interacted with GvpF and GvpL (rf 6.3). Two partner proteins were identified for GvpG (GvpL and GvpF), and GvpL appeared to be the only interaction partner of the three proteins GvpJ, GvpK and GvpM (Figure 3B). Overall, these results obtained by these split-GFP analyses demonstrated that all accessory Gvp proteins had at least one other Gvp protein as interaction partner. Since GvpL bound all of them it is possible that they form a larger complex. Compared to the results obtained by affinity chromatography using _CBD_Gvp, less interactions were observed with split-GFP, especially for the two hydrophobic proteins GvpJ (12 kDa) and GvpM (9.2 kDa), and for GvpK (12.6 kDa).

### Interaction Partner(s) of GvpA

**FIGURE 4:**
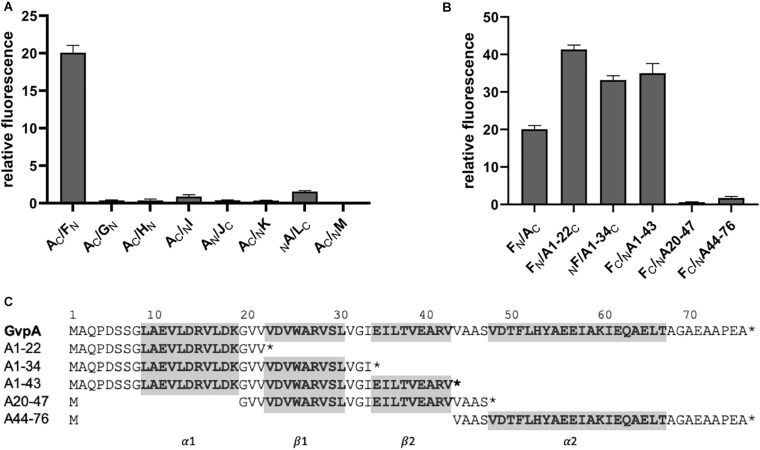
Split-GFP interaction studies with GvpA/GvpX. Only the highest relative fluorescence determined for each combination is given. Two biological and three technical replicates were performed in each case. **(A)** Interaction study of GvpA with the eight accessory proteins GvpF through GvpM. **(B)** Interaction of five different GvpA fragments with GvpF. **(C)** Sequence of the five GvpA fragments in relation to the aa sequence of GvpA shown on top. Numbers above the sequence depict the aa positions in GvpA. The helices α1 and α2 and the β-sheets β1 and β2 are in bold and marked on the bottom and also shaded in gray.

To uncover interactions between the major structural gas vesicle protein GvpA and these accessory Gvp, each combination of A/X (X = GvpF through GvpM) was investigated by split-GFP. GvpA was fused at the N- or C-terminus to N- or CGFP and tested pairwise with the respective N/CGFP fusion variants of GvpF through GvpM. Eight different combinations were investigated for each pair, and the highest rf-values calculated in each case are shown in Figure 4A. The original data obtained in these experiments is presented in Supplementary Figure 6. The highest relative fluorescence was obtained for the A_C_/F_N_ transformant (rf 20), whereas all other transformants yielded rf-values < 1, suggesting very weak contacts between GvpA and the other Gvp proteins (Figure 4A). Except of _N_A/L_C_, the highest fluorescence of a protein pair was always observed when N- or CGFP was fused to the C-terminus of GvpA. These results implied that GvpF is the only interaction partner of GvpA, and that the N-terminal region of GvpA might be involved in the interaction.

To define the interaction site of GvpF in GvpA more precisely, five GvpA fragments harboring different structural features (according to the model obtained by Strunk et al. (2011)) were studied by split-GFP. Fragment A1-22 encompasses the first 22 amino acids of GvpA including α-helix 1 (α1), fragment A1-34 contains in addition β-sheet 1 (α1-β1), and A1-43 the α1-β1-β2 elements of GvpA (Figure 4C). Fragment A20-47 contains β1-β2, and A44-76 the α-helix 2 up to the C-terminus of GvpA (α2) (Figure 4C). Each of these GvpA fragments was fused at the N- or C-terminus to N- or CGFP and tested with the respective N/CGFP fusion of GvpF. Transformant F_N_/A1-22_C_ yielded the highest fluorescence (rf 41), i.e., more than twice as high as obtained for the interaction of GvpF with the entire GvpA (rf 20) (Figure 4B). Smaller protein fragments offer less steric hindrance supporting the interaction (Ghosh et al., 2000; Winter et al., 2018). An increased fluorescence was also found for the transformants harboring F/A1-34 and F/A1-43 (rf 33 – 35), whereas a very low relative fluorescence (rf < 1) was obtained for the transformants harboring F/A20-47 or F/A44-76. These results implied that the C-terminal portion of GvpA is not involved, and that the N-terminal portion contains the interaction site of GvpF.

**FIGURE 5:**
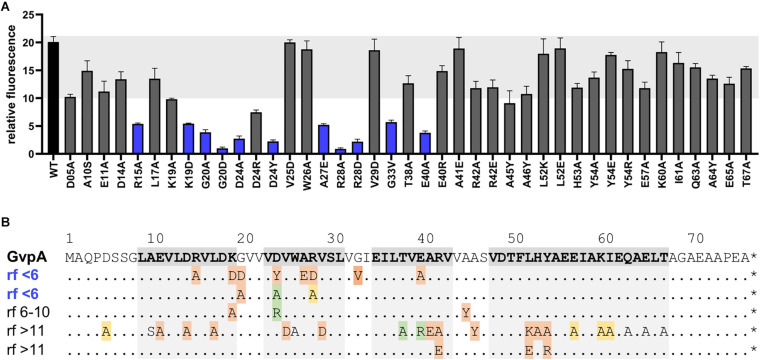
Split-GFP studies of GvpF and variants of GvpA_mut_. **(A)** Rf-values of the GvpF/GvpA (WT) and the various F/A_mut_ transformants. The single substitutions in GvpA are indicated below. F/A_mut_ transformants with relative fluorescence <6 are labeled in blue. The fluorescence was determined in LAU/mm^2^, and the relative fluorescence in relation to the fluorescence of *Hfx. volcanii* wild type calculated. Two biological and three technical replicates were performed in each case. **(B)**. Sequence of GvpA and summary of the rf-values of the various GvpA mutants. The sequence of GvpA is presented on top and the structural features α1–β1–β2–α2 shaded in grey. The substitutions in GvpA are summarized underneath; each GvpA contains only a single substitution. Rf < 6 is regarded as weak interaction, whereas rf > 11 is similar to GvpA wild type. The Vac phenotype of the respective ΔA+A_mut_ transformants is indicated by color: red, Vac negative; green, cylinder shaped; yellow, mini gas vesicles; without color, wild type gas vesicles.

To further confine the interaction site of GvpF in GvpA, various substitution variants of GvpA were tested by split-GFP. Many of these point mutations in GvpA are known to influence the formation of gas vesicles in ΔA+A_mut_ transformants (Strunk et al., 2011; Knitsch et al., 2017). These transformants carry two vector plasmids, the ΔA construct (contains except *gvpA* all *gvp* genes of the p-vac region) and construct A (*gvpA* or mutant *gvpA* expressed in pMDS20 under the control of the native *P*_*A*_ promoter). The different GvpA variants were investigated by split-GFP analysis for their potential to interact with GvpF in *Hfx. volcanii*. The F_N_ fusion protein was used as interaction partner in all these cases (Figure 5A). The strongest reductions in relative fluorescence (rf < 6) compared to GvpA wild type (rf 15-18) were observed for the GvpA substitutions G20D, G20A, D24A, D24Y, R28A, R28D, or E40A, but also R15A, K19D, A27E, or G33V resulted in a low relative fluorescence of the F/A_mut_ transformants (Figure 5A and Supplementary Figure 6). Especially the charged aa in β1 and β2 (D24, R28, E40) and G20 of GvpA had a strong influence on the interaction with GvpF (Figure 5). All these aa are located in the N-terminal half of GvpA, whereas any of the single substitutions in the C-terminal half had no effect on the interaction with GvpF, supporting the results described above. It is likely that these aa of GvpA are involved in the GvpF/GvpA interaction. In respect to the gas vesicle phenotype of the ΔA+A_mut_ transformants, most of these mutations result in a Vac^–^ phenotype, except for D24A (cylindrical) and R28A (mini gas vesicles) (Knitsch et al., 2017; Figure 5B). It is possible that the Vac^–^ phenotype is caused by the lack of an interaction between GvpF and GvpA, rather than (or in addition to) an influence of the mutation on the GvpA/GvpA contact in the gas vesicle wall.

## Discussion

Gas-vesicle assembly involves twelve Gvp proteins, two of which, GvpA and GvpC, form the gas vesicle wall. A third protein, GvpN, is required for the enlargement of the structure, and the function of GvpO is not yet known (Pfeifer, 2012). The accessory proteins GvpF through GvpM are encoded by an operon and produced in minor amounts in early exponential growth. All eight accessory Gvp were investigated pairwise in respect to their interaction(s) and also with the major gas vesicle structural protein, GvpA.

### Pull-Down Experiments Imply Complex Formation of the Accessory Gvp

Earlier pull-down experiments with _His_Gvp proteins indicated non-specific binding of other histidine-rich proteins (such as PitA) to the Ni-NTA matrix, and also non-specific reactions of the Gvp antisera in Western analyses (Tavlaridou et al., 2014, and unpublished observations). In contrast, pull-down assays with _CBD_Gvp on a cellulose matrix yielded a high specificity in the selection. In most cases, single protein bands were detected in the elution fraction, but also multimers were observed, especially with GvpJ, GvpK, and GvpM. None of the Gvp proteins bound to cellulose when the CBD tag was lacking, and the putative binding partner(s) of a given Gvp protein were selected in the respective _CBD_X/Y *Hfx. volcanii* transformants. Earlier studies with _His_M implied an interaction of GvpM with GvpH, GvpJ, and GvpL, but not with GvpG (Tavlaridou et al., 2014). Testing the selection of these Gvp with _CBD_M verified these interactions, but GvpM also interacted with GvpG. A reason for this result could be that the pull-down experiments based on CBD-tagged proteins were performed *in vivo* with transformants synthesizing both bait and prey, whereas the earlier analyses with _His_Gvp were performed *in vitro* with proteins isolated from *E. coli* and refolded in high salt solutions. Only the prey protein was synthesized in *Hfx. volcanii* (Tavlaridou et al., 2014). The larger range of interactions observed with _CBD_X compared to _His_X might be due to the correct folding of bait and prey under *in vivo* conditions. It should be noted that GvpJ and GvpM, but also GvpK oligomerized in the presence of _CBD_I, whereas other _CBD_X selected only monomers and/or dimers of these accessory Gvp. The very positively charged GvpI (pK 10.75) might be able to bind and connect the negatively charged Gvp (pK 4–5). GvpI contains 23 lysine and arginine residues in the N-terminal 53 aa, and the involvement of these aa in the oligomerization of the other proteins and also in the assembly of gas vesicles should be analyzed in further detail.

The results obtained by the pairwise _CBD_X/Y pull-down experiments suggested that all accessory Gvp proteins interact. In almost all cases, the prey proteins were detected by Western analyses. A quantitation of the binding efficiency is difficult, since different antisera were used. A quantitation of the interaction was easier using the split-GFP method and measuring the fluorescence of the cells *in vivo*. The interaction network derived from the pull-down studies suggested that all Gvp proteins attract each other and possibly form a complex. Studies with _CBD_M+pF-L^ex^ transformants demonstrated that GvpM indeed selected all the Gvp proteins at once (Figure 2). It is possible that any of these proteins interacted solely with GvpM, but it is likely that they bound as a complex to the bait. GvpM is required in early steps of gas-vesicle assembly as deduced from studies with ΔM+M_mut_ transformants harboring construct ΔM complemented by GvpM variants with single aa substitutions (Tavlaridou et al., 2014). Only Vac^+^ or Vac^–^ transformants were obtained, whereas variants in gas vesicle shape (as found with many ΔA+A_mut_ transformants) were not observed, implying that GvpM is involved in an early step in gas-vesicle assembly.

### Gvp Interaction Network Deduced by Split-GFP

**FIGURE 6:**
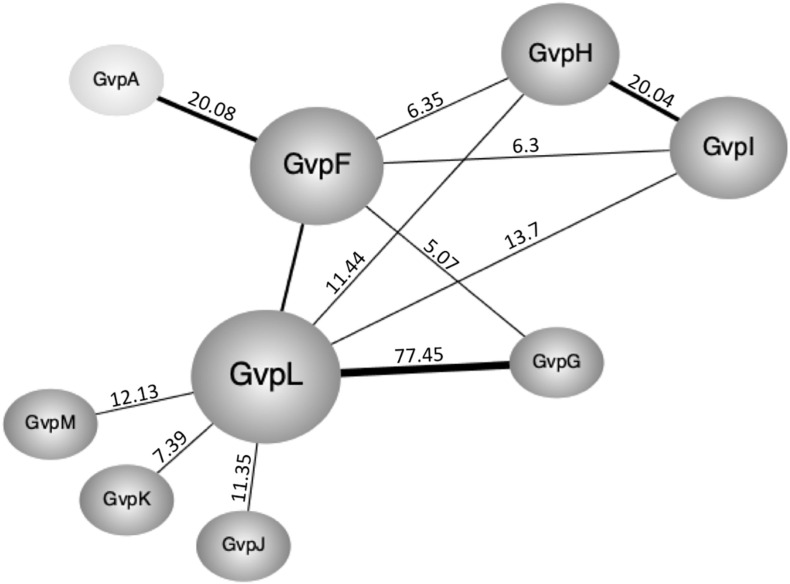
Interaction network of the accessory Gvp and GvpA as obtained by split-GFP. Only clear interactions (rf > 5) are incorporated. The respective rf-values are given at the connecting lines. Rf-values > 20 are indicated by thicker lines.

The split-GFP method is easy to apply and the interactions can be semi-quantified. Each protein pair under investigation requires the analysis of eight combinations to exclude a possible steric hindrance in the assembly of GFP (Winter et al., 2018). Not all of the transformants tested were significantly fluorescent. The calculated relative fluorescence (rf) varied from rf-values < 1, regarded as no protein-protein interaction, over rf 1–5 regarded as weak, to rf > 5 regarded as clear interaction. The data was used to establish a network of the accessory Gvp including all interactions exceeding rf 5 (Figure 6).

The highest number of protein contacts was observed for GvpL that was shown to interact with all other accessory Gvp. The highest rf-value of all combinations tested was found with GvpL/GvpG (rf 77.45). The 10-kDa GvpG is relatively small, similar to GvpJ, GvpK, and GvpM (9.2–12.6 kDa). The latter three proteins yielded GvpL as sole interaction partner, whereas GvpG also contacted GvpF. It is possible that the hydrophobic nature of GvpM and GvpJ and their tendency to aggregate prevented the assembly of sufficient fluorescent GFP in some cases. GvpL (32 kDa) and GvpF (23 kDa) are structurally related as determined by homology modeling using a crystal structure of GvpF derived from the cyanobacterium *Microcystis aeruginosa* as template (Xu et al., 2014; Winter et al., 2018). Since GvpL attracted all other accessory Gvp, this protein might play a central role in the formation of a protein complex. However, it is not known so far whether the binding of the other Gvp occurs at the same time or sequentially.

The interaction partners of GvpF were GvpG, GvpH, GvpI, and GvpL as deduced from the split-GFP analyses (Figures 3, 6). GvpH (19.8 kDa) and GvpI (15.8 kDa) are interesting, since both are non-essential for gas vesicle formation and appear to interact with each other; a relatively high fluorescence (rf 20) was observed for GvpH/GvpI. Both proteins interacted also with GvpL and GvpF. The binding to GvpL resulted in higher rf-values (rf 11.4, GvpH and rf 13.7, GvpI) compared to the rf-values obtained with GvpF (rf 6.3 in both cases) suggesting that the preferred interaction partner of GvpH and GvpI is GvpL. It is possible that GvpH and GvpI form a heterodimer that binds only temporary to the putative complex. GvpH influences the stability of the gas vesicles, whereas a lack of the positively charged GvpI results in extremely long gas vesicles (Offner et al., 2000). Previous studies also suggest that the presence of GvpH prevents the aggregation of GvpM (Tavlaridou et al., 2014). As shown by the pull-down experiments performed in this report, _CBD_H selected GvpM as monomer and not as dimer, whereas all other accessory Gvp selected GvpM additionally as dimer (Figure 1). In contrast, GvpI induced the oligomerization of GvpJ, GvpK, and GvpM (Figure 1). To uncover the exact functions of GvpH and GvpI during gas vesicle formation, both proteins should be investigated in further detail.

Overall, the interaction studies by split-GFP yielded less interactions compared to the pull-down experiments, especially for the hydrophobic GvpJ and GvpM, but also for GvpK. These three proteins often form multimers as observed when investigated by SDS-PAGE. Studies on self-interactions of the Gvp proteins yielded only weak interactions for F/F (rf 1.5), or G/G (rf 3.5), whereas J/J, K/K or M/M transformants were not fluorescent (Supplementary Figure 7). The formation of additional multimers might prevent the formation of fluorescent GFP. These results implied that self-interaction of aggregating proteins is difficult to detect by split-GFP.

### GvpF Is the Only Interaction Partner of GvpA

The analysis of the major gas vesicle structural protein, GvpA, by split-GFP uncovered GvpF as sole binding partner. This result implied that a GvpA/GvpF complex is formed, but since GvpF also attracts GvpG, GvpH, GvpI, and *via* GvpL all other accessory Gvp, it is also possible that a larger complex of the accessory Gvp binds GvpA *via* GvpF. The latter possibility is more likely, since the amount of GvpF is much smaller than the amount of the major gas vesicle protein GvpA. GvpF is only produced in early growth stages (together with GvpG through GvpM), and not in parallel to the massive production of GvpA later in growth. The *P*_*A*_ promoter for the transcription of *gvpA* is 10-fold induced by GvpE, whereas the *P*_*F*_ promoter for the transcription *gvpFGHIJKLM* is less active and not activated by GvpE as determined using GFP as reporter (Born and Pfeifer, 2019). Thus, the GvpF/GvpA binding cannot occur on a 1:1 basis throughout growth, and should happen in early stages of gas vesicle formation.

**FIGURE 7:**
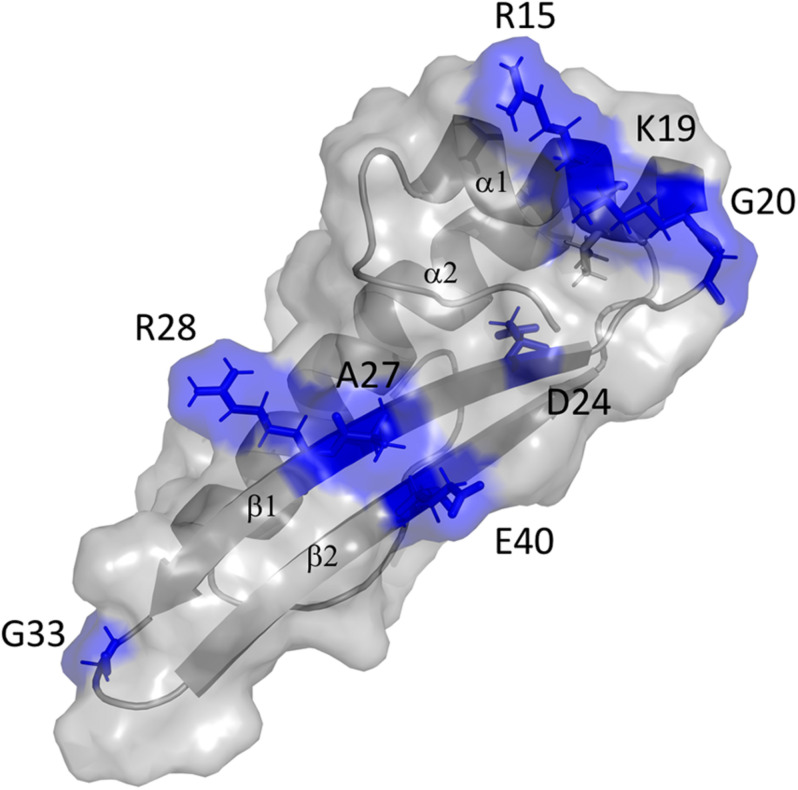
3D-model of GvpA and putative interaction site of GvpF. The GvpA *in silico* model is according to Strunk et al. (2011). The secondary structural features α1, β1, β2, and α2 are labeled. The amino acids marked and colored in blue are positions where a substitution resulted in a low relative fluorescence of the F/A_mut_ transformants.

The contact site of GvpF in GvpA was confined to the first 40 aa of GvpA, since substitutions in the second half of this 76-aa protein had no influence on the GvpA/GvpF interaction. Several single substitutions of amino acids in the N-terminal half of GvpA influenced the GvpA/GvpF interaction, and all these positions are also important for gas vesicle formation (Knitsch et al., 2017). These aa are located in α1, β1 and β2 of GvpA, and the loop regions in between. Except for D24, the charged aa R15, K19, R28, E40 are located on the surface of GvpA (Figure 7; Strunk et al., 2011). These aa are thought to form salt bridges to adjacent GvpA molecules in the gas vesicle wall (Sivertsen et al., 2010; Knitsch et al., 2017), but as shown here they also mediate the GvpA/GvpF contact (when GvpF is available). It is possible that GvpF contacts GvpA prior to the incorporation of GvpA in the gas vesicle wall, to keep this strongly hydrophobic protein in solution (especially by shielding β1-β2) and prevent an undesirable self-aggregation. It is likely that the accessory proteins including GvpF initiate gas vesicle formation by forming a nucleation complex that binds GvpA *via* GvpF and forces GvpA to start aggregating into the ribs of the gas vesicle wall; this hypothesis has been raised already in Shukla and DasSarma (2004).

## Conclusion

A complex (or complexes) of the accessory Gvp proteins might initiate the formation of this gas-filled structure. The aggregation of GvpA to form the ribs of the gas vesicle wall most likely starts at the tips of the two conical end caps of each gas vesicle (Waaland and Branton, 1969), and the small bicones formed are enlarged by further addition of GvpA, and of GvpC at the exterior side. A larger protein complex is required at the tip that binds GvpA to initiate the formation of the gas vesicle wall. The hydrophobic interior surface of ribs formed by GvpA needs to be shielded, especially when the diameter of the conical structure is still small. A complex of the accessory Gvp in both tips could protect and ensure that the ribs become distant enough to expose the hydrophobic surface of GvpA at the interior side and form a hollow structure that rapidly fills with gas. The analysis of the cyanobacterial GvpF by immunogold labeling-based tomography suggests that GvpF is localized at the gas-facing surface of the gas vesicle wall (Xu et al., 2014), supporting this hypothesis. The accessory Gvp are presumably not only required in the tip, but also at the transition of the conical caps into the cylinder structure. In addition, helper proteins might be required at the site where the incorporation of GvpA occurs to enlarge the gas vesicle wall. Presumably this site is located in the center of the gas vesicle, and GvpN might be involved here. All these steps in gas-vesicle assembly are not yet understood, but the accessory Gvp proteins and their complex(es) certainly take part in these processes. Further studies will aim to identify the complex(es) formed by the accessory proteins and determine the location in the gas vesicles structure.

## Data Availability Statement

The original contributions presented in the study are included in the article/Supplementary Material, further inquiries can be directed to the corresponding author.

## Author Contributions

All authors planned the study, discussed the results, wrote the manuscript, and approved the final manuscript. KV and AJ performed the analysis.

## Conflict of Interest

The authors declare that the research was conducted in the absence of any commercial or financial relationships that could be construed as a potential conflict of interest.
