# Supplementary material for: Accessory Gvp Proteins Form a Complex During Gas Vesicle Formation of Haloarchaea
Source: Front Microbiol. 2020 Nov 12;11:610179. doi: 10.3389/fmicb.2020.610179 (PMC7688916; doi:10.3389/fmicb.2020.610179)
Supplement: Supplementary file 1 [file Data_Sheet_1.pdf]

Supplementary Figures

Accessory Gvp Proteins Form a Complex During Gas Vesicle Formation of Haloarchaea

Kerstin Völkner, Alisa Jost and Felicitas Pfeifer

Microbiology and Archaea, Department of Biology, Technical University Darmstadt, Darmstadt, Germany

Supplementary Table 1. Oligonucleotides used in this study.

Name	Oligonucleotide sequence (5‘ – 3‘)*
Split-GFP analysis	
5‘-PciI-pG	tccgag **acatgt** tcatcatagacgatctc
5‘-BamHI-pG	aggtcaa **ggatcc** atgttcatcatagacgatctcttcgtg
3‘-BlpI-pG	tgttgtt **gctcagc** ttatttcttgacctccatgcg
3‘-BlpI-pGΔStop	tgttgtt **gctcagc** ga tttcttgacctccatgcg
3‘-BamHI-pGΔStop	aggtcaa **ggatcc** c tttcttgacctccatgcg
3‘-KpnI-pG	agttct **ggtacc** ttatttcttgacctccatgcgg
5‘-BspHI-pI	tgttgtt **tcatga** gcgacaaacaacagcaaaaacacaag
5‘-BamHI-pI	tgttgtt **ggatcc** atgagcgacaaacaacagcaaaaacac
3‘-BlpI-pI	attc **gctcagc** tcacctcgtcctcagtggg
3‘-BlpI-pIΔStop	tgttgtt **gctcagc** ga ctcatcgttcacctcgtcctc
3‘-BamHI-pIΔStop	aggtcaa **ggatcc** c ctcatcgttcacctcgtcctcag
3‘-KpnI-pI	agttct **ggtacc** tcactcatcgttcacctcgtcctc
5‘-NcoI-pK	acacga **ccatgg** aactagcactcgacgac
5‘-BamHI-pK	aggtcaa **ggatcc** atggaactagcactcgacgacg
3‘-BlpI-pK	tgttgtt **gctcagc** tcatacgtcatcacgctgggattc
3‘-BlpI-pKΔStop	tgttgtt **gctcagc** ga tacgtcatcacgctgggattc
3‘-BamHI-pKΔStop	aggtcaa **ggatcc** c tacgtcatcacgctgggattc
3‘-KpnI-pK	attc **ggtacc** tcatacgtcatcacgctgggattcc
5‘-NcoI-pA	tgttgtt **ccatgg** cgcaaccagattcttc
5‘-BamHI-pA	aggtcaa **ggatcc** atggcgcaaccagattc
3‘-BlpI-pA	tgttgtt **gctcagc** tcaggcctcgggtg
3‘-BlpI-pAΔStop	tgttgtt **gctcagc** ga ggcctcgggtgc
3‘-BamHI-pAΔStop	agttct **ggatcc** c ggcctcgggtg
3‘-KpnI-pA	agttct **ggtacc** tcaggcctcgggtgc
split-GFP – p-gvpA variants	
3’-BlpI-pAa1+Stop	attc **gctcagc** tca aacgacacctttgtctagtacac
3’-BlpI-pAa1	attc **gctcagc** ga aacgacacctttgtctagtacac
3’-BamHI-pA_a1	agttct **ggatcc** c aacgacacctttgtctagtacac
3’-KpnI-pA_a1+Stop	attc **ggtacc** tca aacgacacctttgtctagtacac

5’-NcoI-pA_20-47	attc **ccatgg** gtgtcgttgtggacgtgtg
5’-BamHI-pA_20-47	attc **ggatcc** atg ggtgtcgttgtggacgtg
3’-BlpI-pA_20-47+Stop	attc **gctcagc** tca cgaggcggcgacg
3’-BlpI-pA_20-47	attc **gctcagc** ga cgaggcggcgacg
3’-BamHI-pA_20-47	attc **ggatcc** c cgaggcggcgacg
3’-KpnI-pA_20-47+Stop	attc **ggtacc** tca cgaggcggcgacg
5’-NcoI-pA_a2	attc **ccatgg** tcgccgcctcggtg
5’-BamHI-pA_a2	attc **ggatcc** atg gtcgccgcctcggtg

Pull-down analyses using CBD

5‘-BspHI-pF	acacga **tcatga** ctgagaacctatacacatacggtatcatc
5‘-XbaI-pF	agttct **tctaga** atgactgagaacctatacacatacgg
3‘-BamHI-pF	aggtcaa **ggatcc** cc tcggcctccttgttgctg
3‘-KpnI-pF	agttct **ggtacc** ttatcggcctccttgttgctgttc
5‘-XbaI-pG	agttct **tctaga** atgttcatcatagacgatctcttcg
3‘-KpnI-pG	agttct **ggtacc** ttatttcttgacctccatgcgg
5‘-XbaI-pH	agttct **tctaga** atggttcccgacgaaaacg
3‘-BsrGI-pH	attc **tgtaca** tcatgtggattcacctccatcg
5‘-XbaI-pI	agttct **tctaga** atgagcgacaaacaacagcaaaaac
3‘-KpnI-pI	agttct **ggtacc** tcactcatcgttcacctcgtcctc
5‘-XbaI-pJ	agttct **tctaga** atgagtgaccccaaaccgac
3‘-KpnI-pJ	**ggtacc** tcatttggtctcctccgctgac
5‘-XbaI-pK	agttct **tctaga** atggaactagcactcgacgac
3‘-KpnI-pK	attc **ggtacc** tcatacgtcatcacgctgggattcc
5‘-XbaI-pL	agttct **tctaga** atgact gaccaccggcccag
3‘-KpnI-pL	agttct **ggtacc** ttatttaccaatatctggcgcg
5‘-XbaI-pM	agttct **tctaga** atggagccaacaaaagacgag acac
3‘-KpnI-pM	agttct **ggtacc** tcagtcctctcgccgatc

---

*restriction sites are marked in bold

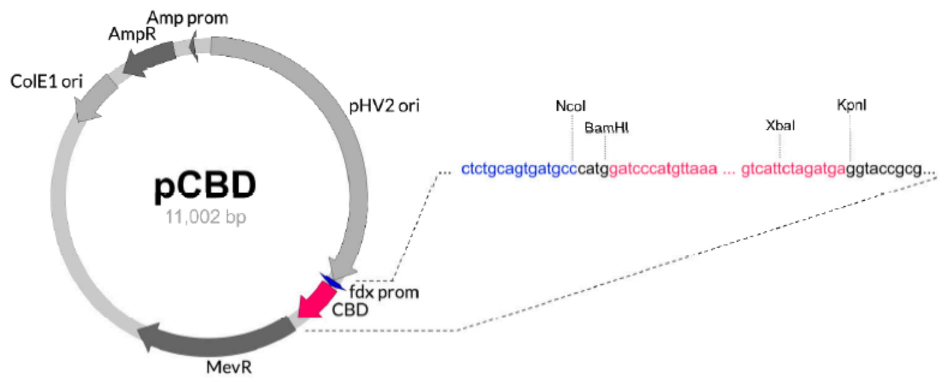

Supplementary Figure 1. Plasmid map of vector pCBD. The expression of ORF inserted is driven by the ferredoxin promoter (fdx prom). The DNA sequences up- and downstream of *cbd* (red arrow and sequence) are shown on the right including the restriction sites used for the fusion of the *gvp* reading frame either at the 5'-end of *cbd* (*Nco*I, *Bam*HI), or at the 3'-end (*Xba*I, *Kpn*I). The shuttle vector contains a mevinolin resistance gene (MevR) for selection in haloarchaea, and an ampicillin resistance gene (AmpR) for selection in *Escherichia coli*. The haloarchaeal origin of replication derives from the *Hfx. volcanii* plasmid pHV2 whereas the ColE1 ori is used in *E. coli*.

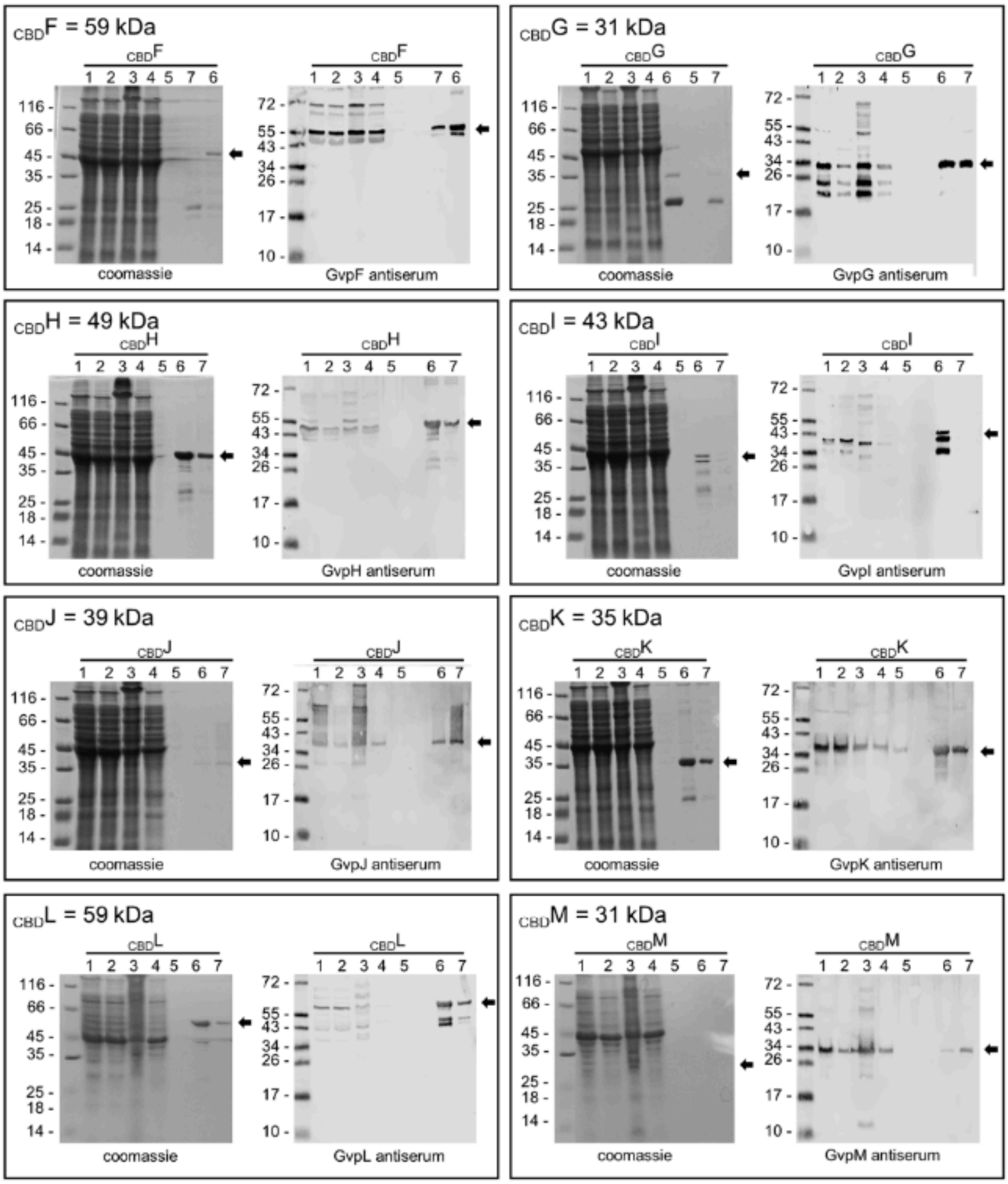

Supplementary Figure 2. Purification of $_{\text{CBD}}$X proteins using a cellulose matrix. In each case, 15 µL of each protein fraction were separated by SDS-PAGE and the proteins stained by Coomassie blue (gel on the left). For Western analysis (blot on the right) the proteins were transferred to a PVDF membrane and incubated with the respective antiserum indicated underneath to detect the protein under investigation. A fluorophore-labelled secondary antibody (IRDye 800CW, Licor) was used for detection. The blots are inverted to black-white. The size markers on the left are in kDa. Arrows mark the position of the expected proteins. 1, cell lysate after sonication; 2, cell lysate after ultracentrifugation; 3, sediment after ultracentrifugation; 4, flow-through; 5, last wash fraction; 6, elution fraction 1; 7, elution fraction 2.

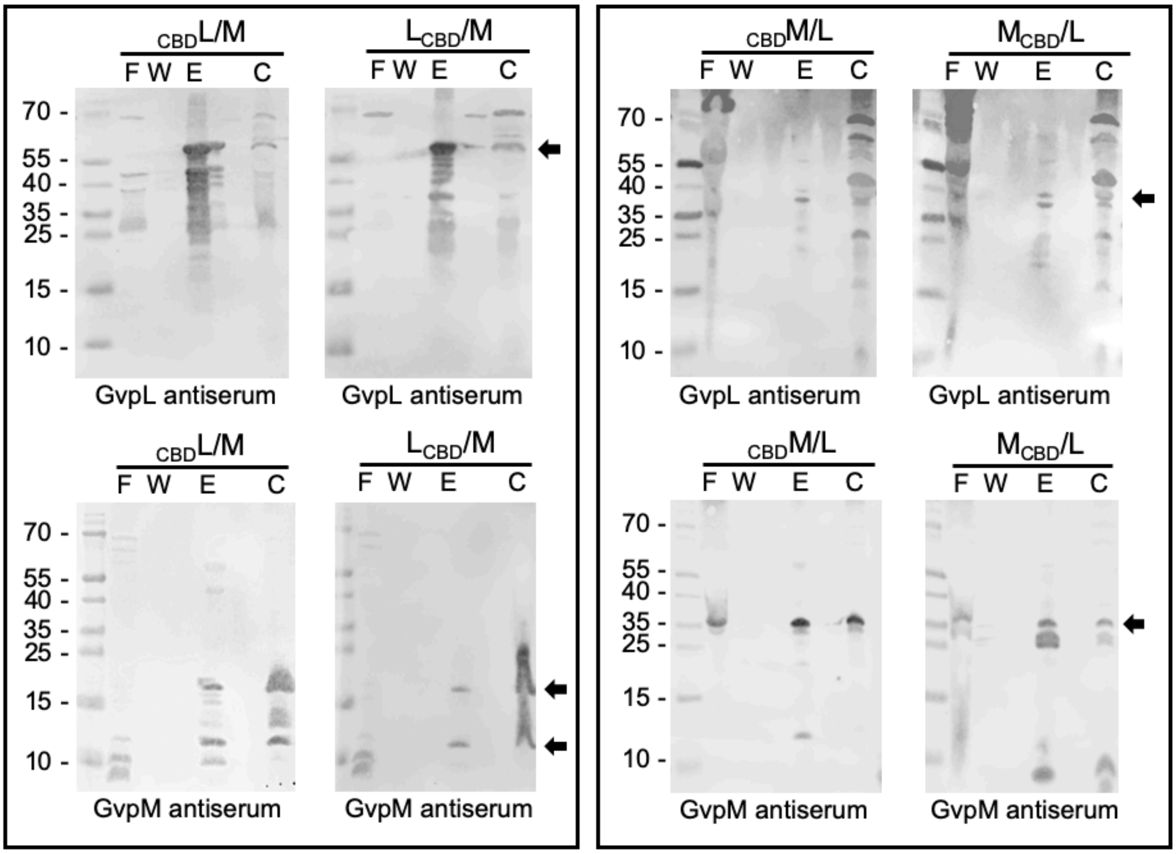

Supplementary Figure 3. Western analyses of pull-down assays to investigate the L/M interaction. The four combinations $_{\text{CBD}}$L/M, L$_{\text{CBD}}$/M, $_{\text{CBD}}$M/L and M$_{\text{CBD}}$/L were analyzed. 20 µg of protein were applied in case of (F), flow-through; and (C), control (= lysate of the respective *Hfx. volcanii* transformant), and 15 µL were applied in the case of the last wash fraction (W) and the elution fraction (E). The proteins were separated by SDS-PAGE, transferred to a PVDF membrane and incubated with the respective Gvp antiserum. The antiserum is marked on the bottom. To visualize the proteins, IRDye 800CW-labelled secondary antisera were used. The expected Gvp monomers (and in case of GvpM also the dimer) are marked by an arrow. The blots are inverted to black and white.

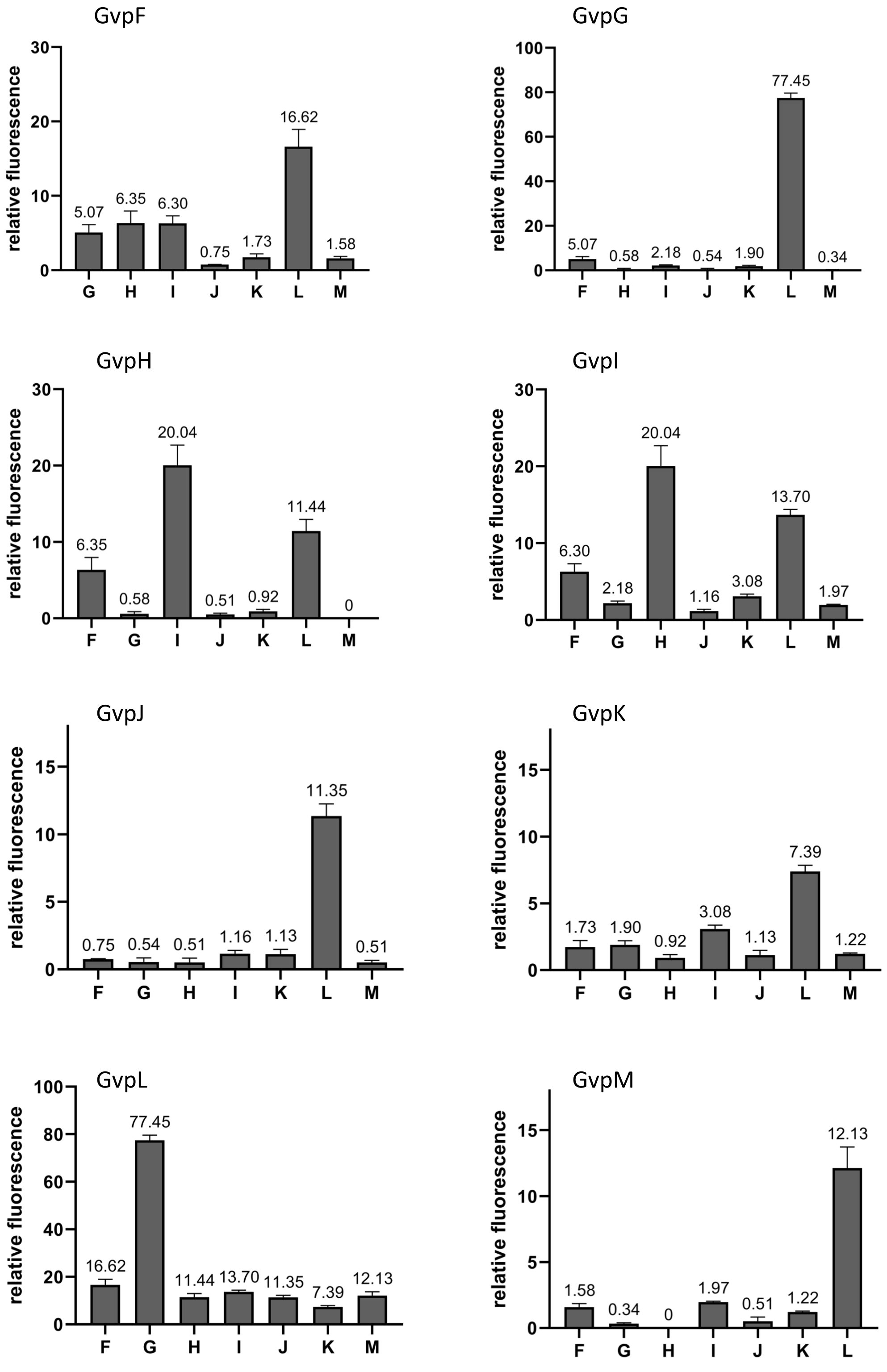

Supplementary Figure 4. Split-GFP analyses to study interactions of the accessory proteins. In each case, only the highest rf-value of the combinations tested is shown. The experimental data underlying these results are presented in supplemental Figure S5. The respective Gvp protein tested is marked on top, and the respective interaction partner on the bottom. The fluorescence was determined in LAU/mm^2. The relative fluorescence, rf, was calculated as indicated in the Material and Methods section. The numbers indicate the highest rf-value determined for each combination. Two biological and three technical replicates were investigated in each case.

transformant		LAU/mm^2	σ (LAU/mm^2)	Relative fluorescence (rf)
**control**				
	WR340	17,066	1,687	0.00
**GvpF interactions**				
$_N$F	G$_C$	87,165	12,609	3.91
	$_C$G	11,884	621	0.00
F$_N$	G$_C$	107,895	17121	1.06
	$_C$G	12,481	1,261	0.00
$_C$F	G$_N$	11,803	391	0.00
	$_N$G	8557	781	0.00
F$_C$	G$_N$	83,692	2,438	0.15
	$_N$G	64,838	5,710	0.35
$_N$F	H$_C$	123,515	24,068	5.95
	$_C$H	11,533	850	0.00
F$_N$	H$_C$	130,511	26,133	6.35
	$_C$H	17,675	1,570	0.04
$_C$F	H$_N$	13,704	869	0.00
	$_N$H	12,312	899	0.00
F$_C$	H$_N$	101,301	8,940	4.70
	$_N$H	73,418	3,017	3.13
$_N$F	I$_C$	109,826	16,264	5.10
	$_C$I	11,082	555	0.00
F$_N$	I$_C$	129,765	16,305	6.30
	$_C$I	10,787	909	0.00
$_C$F	I$_N$	10,213	2,586	0.00
	$_N$I	10,532	881	0.00
F$_C$	I$_N$	112,863	4,033	5.35
	$_N$I	127,148	9,485	6.16
$_N$F	J$_C$	28,027	565	0.75
	$_C$J	9,612	1,087	0.00
F$_N$	J$_C$	24,642	943	0.54
	$_C$J	9,073	1,136	0.00
$_C$F	J$_N$	11,566	402	0.00
	$_N$J	8,361	1,287	0.00
F$_C$	J$_N$	13,853	1,428	0.00
	$_N$J	15,429	2,314	0.05
$_N$F	K$_C$	32,122	4,565	1.01
	$_C$K	10,029	1,748	0.00
F$_N$	K$_C$	38,081	13,976	1.38
	$_C$K	8,776	1,761	0.00
$_C$F	K$_N$	9,564	217	0.00
	$_N$K	7,647	1,932	0.00
F$_C$	K$_N$	13,826	390	0.00
	$_N$K	43,619	7,062	1.73
$_N$F	L$_C$	181,946	12,207	10.65
	$_C$L	16,733	1,502	0.08
F$_N$	L$_C$	210,293	4,889	12.51
	$_C$L	21,354	2,026	0.36
$_C$F	L$_N$	15,145	1,594	0.05
	$_N$L	24,034	5,085	0.53
F$_C$	L$_N$	39,902	1,176	1.57
	$_N$L	273,631	15,616	16.62

transformant		LAU/mm^2	σ (LAU/mm^2)	Relative fluorescence (rf)
**GvpG interactions**				
$_N$G	H$_C$	17,038	2,091	0.10
	$_C$H	10,528	1,983	0.00
G$_N$	H$_C$	20,611	3,106	0.30
	$_C$H	9,237	1,080	0.00
$_C$G	H$_N$	10,196	2,690	0.00
	$_N$H	10,583	1,526	0.00
G$_C$	H$_N$	25,209	4,254	0.58
	$_N$H	21,067	1,111	0.32
$_N$G	I$_C$	35,901	1,329	0.72
	$_C$I	16,309	1,628	0.00
G$_N$	I$_C$	40,554	1,630	0.95
	$_C$I	16,155	2,636	0.00
$_C$G	I$_N$	18,190	636	0.00
	$_N$I	15,385	334	0.00
G$_C$	I$_N$	41,093	5,334	0.97
	$_N$I	66,154	5,393	2.18
$_N$G	J$_C$	26,399	3,803	0.27
	$_C$J	13,521	2,029	0.00
G$_N$	J$_C$	32,074	5,853	0.54
	$_C$J	12,786	1,918	0.00
$_C$G	J$_N$	12,732	2,657	0.00
	$_N$J	12,236	1,238	0.00
G$_C$	J$_N$	16,635	2,669	0.00
	$_N$J	18,960	4,425	0.06
$_N$G	K$_C$	24,821	4,076	0.23
	$_C$K	15,038	1,402	0.00
G$_N$	K$_C$	21,793	3,924	0.11
	$_C$K	13,127	1,348	0.00
$_C$G	K$_N$	15,293	2,526	0.00
	$_N$K	17,784	2,230	0.00
G$_C$	K$_N$	19,943	1,215	0.01
	$_N$K	60,428	5,626	1.90
$_N$G	L$_C$	35,575	718	1.25
	$_C$L	1,239,724	36,995	77.45
G$_N$	L$_C$	19,318	1,256	0.22
	$_C$L	291,817	42,477	17.47
$_C$G	L$_N$	31,982	2,466	1.02
	$_N$L	39,238	2,815	1.48
G$_C$	L$_N$	90,246	11,496	4.71
	$_N$L	429,801	30,057	26.20
$_N$G	M$_C$	13,228	838	0.01
	$_C$M	12,356	657	0.00
G$_N$	M$_C$	18,243	3,416	0.31
	$_C$M	12,417	947	0.00
$_C$G	M$_N$	11,020	1,717	0.00
	$_N$M	10,889	447	0.00
G$_C$	M$_N$	18,661	581	0.34
	$_N$M	16,982	1,264	0.22

continued

transformant		LAU/mm^2	σ (LAU/mm^2)	Relative fluorescence (rf)
**GvpH interactions**				
$_N$H	I$_C$	79,625	4,381	4.04
	$_C$I	15,828	1,533	0.05
H$_N$	I$_C$	111,516	9,401	6.06
	$_C$I	15,342	441	0.00
$_C$H	I$_N$	17,990	1,491	0.14
	$_N$I	17,653	1,952	0.13
H$_C$	I$_N$	215,399	7,709	12.63
	$_N$I	332,556	38,157	20.04
$_N$H	J$_C$	20,514	4,651	0.30
	$_C$J	13,485	947	0.00
H$_N$	J$_C$	23,868	2,257	0.51
	$_C$J	15,275	2,800	0.07
$_C$H	J$_N$	12,442	1,006	0.00
	$_N$J	12,598	737	0.00
H$_C$	J$_N$	16,434	2,392	0.09
	$_N$J	17,174	1,350	0.09
$_N$H	K$_C$	28,049	2,887	0.28
	$_C$K	14,560	580	0.00
H$_N$	K$_C$	28,790	4,181	0.31
	$_C$K	14,470	1,760	0.00
$_C$H	K$_N$	14,173	1,091	0.00
	$_N$K	14,915	715	0.00
H$_C$	K$_N$	17,426	1,006	0.00
	$_N$K	42,257	4,777	0.92
$_N$H	L$_C$	202,765	12,468	8.23
	$_C$L	18,806	883	0.00
H$_N$	L$_C$	273,308	30,464	11.44
	$_C$L	20,533	1,778	0.01
$_C$H	L$_N$	14,659	2,056	0.00
	$_N$L	21,167	923	0.01
H$_C$	L$_N$	46,774	7,154	1.13
	$_N$L	267,281	38,757	11.17
**GvpI interactions**				
$_N$I	J$_C$	47,459	4,718	1.16
	$_C$J	18,000	2,048	0.00
I$_N$	J$_C$	42,790	11,595	0.95
	$_C$J	15,340	2,257	0.00
$_C$I	J$_N$	16,641	2,873	0.01
	$_N$J	16,523	3,415	0.00
I$_C$	J$_N$	20,869	976	0.00
	$_N$J	28,206	5,405	0.28
$_N$I	K$_C$	73,778	4,705	3.08
	$_C$K	15,528	2,365	0.01
I$_N$	K$_C$	67,321	2,123	2.73
	$_C$K	13,646	7,988	0.00
$_C$I	K$_N$	12,086	719	0.00
	$_N$K	13,526	708	0.00
I$_C$	K$_N$	15,761	1,798	0.00
	$_N$K	14,970	2,339	0.00

transformant		LAU/mm^2	σ (LAU/mm^2)	Relative fluorescence (rf)
$_N$I	L$_C$	265,687	11,216	13.70
	$_C$L	27,854	1,437	0.54
I$_N$	L$_C$	217,188	20,733	11.02
	$_C$L	19,779	1,831	0.11
$_C$I	L$_N$	15,057	963	0.00
	$_N$L	24,251	2,168	0.34
I$_C$	L$_N$	30,278	3,556	0.68
	$_N$L	14,127	1,057	0.00
$_N$I	M$_C$	9,736	747	0.00
	$_C$M	10,929	469	0.00
I$_N$	M$_C$	9,315	436	0.00
	$_C$M	10,898	762	0.00
$_C$I	M$_N$	10,807	456	0.00
	$_N$M	11,605	747	0.00
I$_C$	M$_N$	25874	4,482	0.71
	$_N$M	44,890	890	1.97
**GvpJ interactions**				
$_N$J	K$_C$	20,329	1,763	0.12
	$_C$K	13,329	474	0.00
J$_N$	K$_C$	15,603	4,395	0.06
	$_C$K	13,848	562	0.00
$_C$J	K$_N$	15,275	1,174	0.00
	$_N$K	18,514	5,587	0.11
J$_C$	K$_N$	19,308	1,414	0.07
	$_N$K	38,556	5,618	1.13
$_N$J	L$_C$	45,961	2,668	1.20
	$_C$L	15,806	1,480	0.00
J$_N$	L$_C$	57,894	814	1.77
	$_C$L	15,955	2,645	0.00
$_C$J	L$_N$	13,896	707	0.00
	$_N$L	28,710	7,991	0.37
J$_C$	L$_N$	80,039	64,248	2.83
	$_N$L	258,153	17,047	11.35
**GvpK interactions**				
$_N$K	L$_C$	161,909	12,313	6.75
	$_C$L	18,642	577	0.00
K$_N$	L$_C$	43,234	3,570	1.07
	$_C$L	13,152	643	0.00
$_C$K	L$_N$	13,565	3,427	0.00
	$_N$L	21,653	502	0.04
K$_C$	L$_N$	58,646	5,108	1.81
	$_N$L	175,356	8,856	7.39
$_N$K	M$_C$	14,160	601	0.17
	$_C$M	11,496	909	0.01
K$_N$	M$_C$	13,762	599	0.14
	$_C$M	10,704	500	0.00
$_C$K	M$_N$	9,237	1,112	0.00
	$_N$M	12,428	1,471	0.07
K$_C$	M$_N$	14,730	752	0.22
	$_N$M	26,819	717	1.22

Supplementary Figure 5. Split-GFP analyses investigating pairwise interactions of the accessory Gvp. The fluorescence was measured in LAU/mm^2 and the relative fluorescence was calculated according to Winter et al. (2018). The GvpL and GvpM interactions have been published already (Winter et al., 2018).

GvpA interactions

transformant		LAU/mm^2	σ (LAU/mm^2)	Relative fluorescence (rf)
$_{N}A$	F_{C}	80,483	8,502	2.26
	$_{C}F$	20,992	2,000	0.00
A_{N}	F_{C}	339,947	103,435	12.70
	$_{C}F$	21,221	1,561	0.00
$_{C}A$	F_{N}	22,051	514	0.00
	$_{N}F$	18,923	1,697	0.00
A_{C}	F_{N}	519,408	8,655	20.08
	$_{N}F$	307,815	14,082	11.49
$_{N}A$	G_{C}	25,165	1,811	0.04
	$_{C}G$	17,403	1,423	0.00
A_{N}	G_{C}	24,115	2,508	0.03
	$_{C}G$	18,958	2,036	0.00
$_{C}A$	G_{N}	19,055	1,491	0.00
	$_{N}G$	17,347	1,935	0.00
A_{C}	G_{N}	33,778	2,061	0.37
	$_{N}G$	23,833	585	0.00
$_{N}A$	H_{C}	29,749	4,899	0.21
	$_{C}H$	19,855	2,103	0.00
A_{N}	H_{C}	24,780	2,196	0.03
	$_{C}H$	18,986	756	0.00
$_{C}A$	H_{N}	19,831	2,188	0.00
	$_{N}H$	18,922	811	0.00
A_{C}	H_{N}	33,569	4,202	0.36
	$_{N}H$	21,477	2,089	0.00
$_{N}A$	I_{C}	33,547	2,397	0.70
	$_{C}I$	19,385	888	0.03
A_{N}	I_{C}	24,592	1,373	0.25
	$_{C}I$	20,112	548	0.04
$_{C}A$	I_{N}	19,994	767	0.02
	$_{N}I$	19,541	833	0.02
A_{C}	I_{N}	32,643	1,295	0.65
	$_{N}I$	37,083	3,425	0.88
$_{N}A$	J_{C}	25,476	1,182	0.29
	$_{C}J$	20,220	2,845	0.10
A_{N}	J_{C}	27,322	615	0.38
	$_{C}J$	21,663	703	0.10
$_{C}A$	J_{N}	21,452	571	0.09
	$_{N}J$	22,067	2,247	0.14
A_{C}	J_{N}	26,490	1,987	0.35
	$_{N}J$	21,987	863	0.11
$_{N}A$	K_{C}	24,203	3,686	0.22
	$_{C}K$	18,545	1,584	0.03
A_{N}	K_{C}	20,151	1,560	0.04
	$_{C}K$	18,825	2,477	0.06
$_{C}A$	K_{N}	20,411	2,154	0.06
	$_{N}K$	20,693	1,826	0.09
A_{C}	K_{N}	23,525	2,914	0.20
	$_{N}K$	26,653	613	0.35
$_{N}A$	L_{C}	60,119	1,419	1.55
	$_{C}L$	18,841	1,281	0.00
A_{N}	L_{C}	27,252	1,212	0.16
	$_{C}L$	18,823	1,727	0.00
$_{C}A$	L_{N}	19,369	1,399	0.00
	$_{N}L$	17,840	1,547	0.00
A_{C}	L_{N}	21,534	673	0.00
	$_{N}L$	58,279	3,713	1.47
$_{N}A$	M_{C}	17,676	1,043	0.00
	$_{C}M$	14,852	1,822	0.00
A_{N}	M_{C}	17,103	940	0.00
	$_{C}M$	13,804	2,094	0.00
$_{C}A$	M_{N}	16,728	442	0.00
	$_{N}M$	13,321	3,229	0.00
A_{C}	M_{N}	16,873	768	0.00
	$_{N}M$	19,676	738	0.00

GvpF/GvpA variant interactions

transformant		LAU/mm^2	σ (LAU/mm^2)	Relative fluorescence (rf)
$_{N}F$	A1-22$_{C}$	883,501	17,024	32.18
	$_{C}$A1-22	18,199	604	0.00
F_{N}	A1-22$_{C}$	1,128,200	27,676	41.37
	$_{C}$A1-22	17,209	739	0.00
$_{C}F$	A1-22$_{N}$	30,056	3,750	0.14
	$_{N}$A1-22	28,858	737	0.10
F_{C}	A1-22$_{N}$	289,486	100,816	9.87
	$_{N}$A1-22	276,399	23,581	9.38
$_{N}F$	A1-34$_{C}$	900,212	28,863	33.17
	$_{C}$A1-34	20,460	1,604	0.00
F_{N}	A1-34$_{C}$	815,906	63,597	29.97
	$_{C}$A1-34	21,191	652	0.00
$_{C}F$	A1-34$_{N}$	34,939	3,383	0.33
	$_{N}$A1-34	34,686	2,653	0.32
F_{C}	A1-34$_{N}$	714,437	58,861	26.12
	$_{N}$A1-34	574,039	54,316	20.79
$_{N}F$	A1-43$_{C}$	414,464	30,090	14.73
	$_{C}$A1-43	30,311	683	0.15
F_{N}	A1-43$_{C}$	621,524	30,005	22.59
	$_{C}$A1-43	31,363	1,009	0.19
$_{C}F$	A1-43$_{N}$	26,879	976	0.00
	$_{N}$A1-43	41,467	3,329	0.41
F_{C}	A1-43$_{N}$	441,419	37,389	14.04
	$_{N}$A1-43	949,021	61,828	35.02
$_{N}F$	A20-47$_{C}$	17,076	1,178	0.02
	$_{C}$A20-47	14,138	833	0.00
F_{N}	A20-47$_{C}$	18,422	428	0.08
	$_{C}$A20-47	17,691	1,027	0.00
$_{C}F$	A20-47$_{N}$	17,002	1,769	0.01
	$_{N}$A20-47	15,326	1,128	0.00
F_{C}	A20-47$_{N}$	19,097	1,239	0.03
	$_{N}$A20-47	30,382	1,800	0.61

continued

transformant		LAU/mm^2	σ (LAU/mm^2)	Relative fluorescence (rf)
$_N$F	A44-76$_C$	27,008	1,236	0.33
	$_C$A44-76	18,335	1,202	0.00
F$_N$	A44-76$_C$	29,068	1,381	0.44
	$_C$A44-76	18,231	514	0.00
$_C$F	A44-76$_N$	20,533	900	0.03
	$_N$A44-76	23,737	2,279	0.17
F$_C$	A44-76$_N$	33,339	1,651	0.65
	$_N$A44-76	55,334	7,966	1.73
F$_N$	A_D05A$_C$	145,274	5,969	10.23
	A_A10S$_C$	205,825	23,292	14.91
	A_E11A$_C$	149,443	22,841	11.18
	A_D14A$_C$	176,443	16,511	13.38
	A_R15A$_C$	78,324	2,011	5.38
	A_L17A$_C$	173,576	22,122	13.50
	A_K19A$_C$	129,419	2,041	9.81
	A_K19D$_C$	76,832	1,249	5.42
	A_G20A$_C$	58,340	5,496	3.87
	A_G20D$_C$	23,935	2,689	1.00
	A_D24A$_C$	48,123	6,107	2.72
	A_D24R$_C$	109,337	5,458	7.45
	A_D24Y$_C$	38,610	3,054	2.23
	A_V25D$_C$	251,534	5,568	20.01
	A_W26A$_C$	255,805	19,554	18.77
	A_A27E$_C$	74,385	2,394	5.21
	A_R28A$_C$	22,957	1,937	0.89
	A_R28D$_C$	38,357	5,084	2.20
	A_V29D$_C$	234,713	23,767	18.61
	A_G33V$_C$	76,586	4,021	5.70
	A_T38A$_C$	166,067	16,901	12.65
	A_E40A$_C$	54,640	3,681	3.78
	A_E40R$_C$	205,234	12,382	14.86
	A_A41E$_C$	227,454	22,677	18.91
	A_R42A$_C$	146,180	13,993	11.80
	A_R42E$_C$	147,994	14,863	11.95
	A_A45Y$_C$	115,337	25,230	9.10
	A_A46Y$_C$	134,355	15,617	10.76
	A_L52K$_C$	216,712	30,479	17.97
	A_L52E$_C$	227,712	21,544	18.93
	A_H53A$_C$	147,257	8,522	11.89
	A_Y54A$_C$	190,128	12,925	13.69
	A_Y54E$_C$	242,711	5,871	17.76
	A_Y54R$_C$	210,400	18,905	15.26
	A_E57A$_C$	155,862	13,073	11.81
	A_K60A$_C$	234,490	22,164	18.27
	A_I61A$_C$	210,754	22,921	16.32
	A_Q63A$_C$	201,183	8,315	15.53
	A_A64Y$_C$	176,626	7,399	13.51
	A_E65A$_C$	165,484	14,097	12.60
	A_T67A$_C$	198,859	4,119	15.34

Supplementary Figure 6. Split-GFP analyses to study the interaction of GvpA (wild type and variants) with GvpF. The fluorescence was measured in LAU/mm^2 and the relative fluorescence was calculated according to Winter et al. (2018).

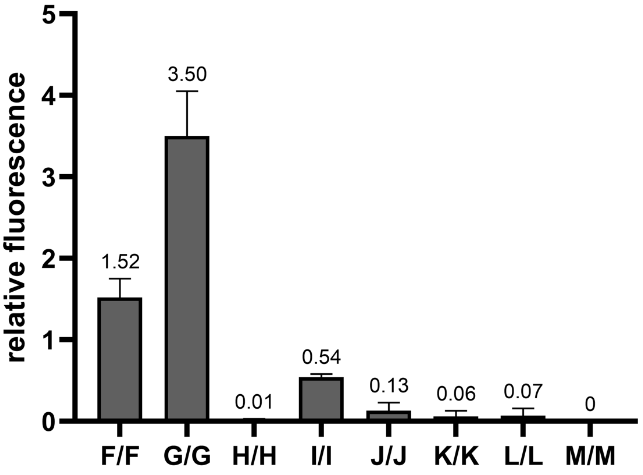

Supplementary Figure 7. Split-GFP analyses of the self-interaction of the accessory Gvp. The experimental data underlying these results are presented in supplemental Figure S8. The fluorescence was determined in LAU/mm^{2}, and the rf-value was calculated. The numbers indicate the highest rf-value determined for each combination. Two biological and three technical replicates were investigated in each case.

transformant		LAU/mm^2	σ (LAU/mm^2)	Relative fluorescence (rf)	transformant		LAU/mm^2	σ (LAU/mm^2)	Relative fluorescence (rf)
$_N$F	F$_C$	25,436	1,600	0.62	$_N$J	J$_C$	22,909	1,095	0.09
	$_C$F	26,551	2,647	0.69		$_C$J	23,675	2,053	0.13
F$_N$	F$_C$	24,283	7,001	0.54	J$_N$	J$_C$	20,952	1,751	0.03
	$_C$F	39,650	3,693	1.52		$_C$J	23,908	2,478	0.13
$_N$G	G$_C$	24,703	2,459	0.57	$_N$K	K$_C$	21,527	1,432	0.04
	$_C$G	34,010	2,683	1.16		$_C$K	21,520	876	0.03
G$_N$	G$_C$	62,546	7,618	3.50	K$_N$	K$_C$	18,464	928	0.00
	$_C$G	31,379	5,645	0.99		$_C$K	21,915	1,8748	0.06
$_N$H	H$_C$	15,347	1,028	0.00	$_N$L	L$_C$	21,801	2,655	0.07
	$_C$H	13,819	481	0.00		$_C$L	17,791	2,033	0.02
H$_N$	H$_C$	11,913	1,197	0.00	L$_N$	L$_C$	17,853	1,242	0.00
	$_C$H	15,227	1,023	0.01		$_C$L	18,938	1,212	0.01
$_N$I	I$_C$	20,631	492	0.54	$_N$M	M$_C$	14,432	1,312	0.00
	$_C$I	12,156	3,478	0.08		$_C$M	12,406	936	0.00
I$_N$	I$_C$	15,811	281	0.18	M$_N$	M$_C$	14,378	2,175	0.00
	$_C$I	16,820	1,166	0.26		$_C$M	12,805	835	0.00

Supplementary Figure 8. Split-GFP analyses to study the self-interaction of the different accessory Gvp proteins. The fluorescence was measured in LAU/mm^2 and the relative fluorescence calculated.
